# Shoaling behaviour in response to turbidity in three‐spined sticklebacks

**DOI:** 10.1002/ece3.10708

**Published:** 2023-11-07

**Authors:** Hannah E. A. MacGregor, Christos C. Ioannou

**Affiliations:** ^1^ Department of Zoology University of Cambridge Cambridge UK; ^2^ School of Biological Sciences University of Bristol Bristol UK

**Keywords:** anthropogenic change, collective behaviour, collective motion, environmental change, *Gasterosteus aculeatus*, vision

## Abstract

Many fresh and coastal waters are becoming increasingly turbid because of human activities, which may disrupt the visually mediated behaviours of aquatic organisms. Shoaling fish typically depend on vision to maintain collective behaviour, which has a range of benefits including protection from predators, enhanced foraging efficiency and access to mates. Previous studies of the effects of turbidity on shoaling behaviour have focussed on changes to nearest neighbour distance and average group‐level behaviours. Here, we investigated whether and how experimental shoals of three‐spined sticklebacks (*Gasterosteus aculeatus*) in clear (<10 Nephelometric Turbidity Units [NTU]) and turbid (~35 NTU) conditions differed in five local‐level behaviours of individuals (nearest and furthest neighbour distance, heading difference with nearest neighbour, bearing angle to nearest neighbour and swimming speed). These variables are important for the emergent group‐level properties of shoaling behaviour. We found an indirect effect of turbidity on nearest neighbour distances driven by a reduction in swimming speed, and a direct effect of turbidity which increased variability in furthest neighbour distances. In contrast, the alignment and relative position of individuals was not significantly altered in turbid compared to clear conditions. Overall, our results suggest that the shoals were usually robust to adverse effects of turbidity on collective behaviour, but group cohesion was occasionally lost during periods of instability.

## Graphical abstract

Turbidity, the scattering of light by suspended particles in water, can severely impair the ability of aquatic animals to use visual cues. Here we investigated how experimental shoals of three‐spined sticklebacks (*Gasterosteus aculeatus*) responded to the sensory challenge of turbidity. Our results suggest that the shoals were usually robust to adverse effects of turbidity on shoaling, but group cohesion was occasionally lost during periods of instability.

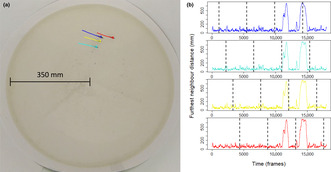

## 1 INTRODUCTION

Turbidity, the scattering of light by suspended particles in water, can severely impair the ability of aquatic animals to use visual cues as sources of private or social information (Chamberlain & Ioannou, 2019; Utne‐Palm, 2002). For group‐living species such as shoaling fish, this can be particularly problematic because they frequently use vision for social information transfer during their collective behaviour (Couzin et al., 2002; Giardina, 2008; Herbert‐Read, 2016). Social information in fish shoals is typically acquired passively and from their local vicinity as individuals respond to visual and mechanosensory cues on the position, orientation and movement of their near neighbours (Ioannou, 2017). Attraction and alignment between neighbours that maintain cohesion and coordination during shoaling typically depend on the visual capabilities of individuals in groups (Kowalko et al., 2013; Partridge & Pitcher, 1980; Strandburg‐Peshkin et al., 2013), whereas other sensory modalities are thought to be more important for repulsion and collision avoidance (e.g. the lateral line, Faucher et al., 2010). The maintenance of cohesion and coordination during shoaling is essential for many of the key benefits of group living such as predator avoidance (Magurran, 1990) and enhanced foraging (Pitcher et al., 1982; Ryer & Olla, 1991). How individuals in groups behave in response to elevated turbidity is therefore likely to be crucial for their survival in turbid environments.

Coastal and freshwater habitats often experience natural fluctuations in turbidity seasonally and with changing weather conditions. Plasticity in behaviour and sensory systems could allow shoaling fish to overcome the challenge of maintaining collective behaviour during periods of elevated turbidity in these environments, just as plasticity in mating effort has been shown to compensate for negative effects on mating in increased turbidity (Ehlman et al., 2018). For example, fish may modify their behaviour to improve the perception of social information (e.g. swim closer to their neighbours) or shift their reliance to alternative sensory modalities less sensitive to turbidity (e.g. the lateral line and chemical cues). Of increasing concern are anthropogenic increases in turbidity in aquatic environments due to climate change and activities such as agriculture and urbanisation (Chapman et al., 2014; Davies‐Colley & Smith, 2001; Mi et al., 2019; Smith, 2003). Here, fish may lack the sensory and behavioural capabilities to respond adaptively to levels of turbidity outside the normal range. Although fish shoals may be robust to small increases in turbidity (Allibhai et al., 2023; Zanghi et al., 2023), several experimental studies have found that more extreme short‐term increases in turbidity can lead to a breakdown in shoaling behaviour, with some evidence that this is due to mechanistic constraints on the availability of visual cues rather than as an adaptive response (Borner et al., 2015; Chamberlain & Ioannou, 2019; Fischer & Frommen, 2013; Kimbell & Morrell, 2015b; Michael et al., 2021). Even small changes in collective behaviour could affect the adaptive benefits for shoaling fish (Ioannou et al., 2019; Romenskyy et al., 2020), with detrimental effects on the survival and reproduction of individuals. This could help to explain changes in the structure of freshwater fish communities in response to anthropogenic increases in turbidity (Berkman & Rabeni, 1987; Kemp et al., 2011).

Here, we tested how groups of wild freshwater three‐spined sticklebacks (*Gasterosteus aculeatus*) from a UK population altered their behaviour in response to the sensory challenge of turbidity. 19 groups of four individuals were filmed in an open arena on the riverbank at their site of capture under both clear and turbid conditions to better understand the impact of turbidity on four measures of collective behaviour (nearest and furthest neighbour distances, heading difference with nearest neighbour and bearing angle of nearest neighbour). The nearest neighbour distance is a measure of how the local interactions between fish are affected by turbidity, while the furthest neighbour distance is a measure of cohesion for the whole group. The difference in heading of the nearest neighbour can reveal whether turbidity is affecting the directional alignment between neighbours and hence coordination. The bearing angle of the nearest neighbour is a measure of group structure, that is how individuals are positioned relative to one another. We also included swimming speed in the analyses as previous work has demonstrated its importance in fish collective behaviour, where swimming speed is associated with longer neighbour distance and increased polarisation (i.e. smaller differences in heading between fish) (Herbert‐Read et al., 2011; Tunstrøm et al., 2013).

Previous experimental studies examining the effects of turbidity on shoaling behaviour have used laboratory‐bred or wild‐caught fish held in captivity and have typically focussed on nearest neighbour distance and average group‐level traits (e.g. Borner et al., 2015; Michael et al., 2021). Here, we used high‐resolution video tracking that allows for the collection of detailed spatio‐temporal information to quantify other key aspects of shoaling behaviour. We focussed on behaviours at an individual level rather than average group‐level traits (which includes measures such as polarisation, convex hull area and group centroid speed) because it is changes to these local interactions that will influence shoaling behaviour in turbid water. We conducted our experiment on the riverbank rather than in the laboratory to capture behavioural responses to turbidity that were unaffected by the potential influences of transportation to laboratory facilities and captive housing. This also had the advantage that the fish could be released back into the wild at their point of capture on the same day. Our overall aim was to contribute a more detailed understanding of shoaling responses to turbidity than previously reported.

Based on previous studies of shoaling fish, we predicted that the turbidity treatment would lead to a breakdown in cohesion and coordination in the groups due to a lack of reliable visual information, with increased nearest and furthest neighbour distances and greater heading difference with nearest neighbours. However, if adaptive plastic responses to turbidity are able to maintain cohesion and coordination, we expected to find the lack of change in neighbour distances and/or group structure (neighbour heading difference and bearing) may be associated with a reduction in swimming speed. Alternatively, if elevated turbidity does not affect the ability of the fish to gather adequate visual information to maintain collective behaviour, or if they are able to compensate by increasing reliance on non‐visual sensory modalities, we would not expect to find any differences in the shoaling traits between the treatments.

## 2 MATERIALS AND METHODS

### 2.1 Ethics and permits

This research was approved by the University of Bristol as a non‐project licence investigation involving animals (UIN/17/060). Fish were captured with the permission of the Environment Agency (permit number EP/EW066‐D‐511/20505/01).

### 2.2 Study site and animals

Three‐spined sticklebacks (standard body length: 37 ± 3.3 mm, mean ± SD) were caught from the River Cary at Somerton, Somerset (Grid reference: ST47813068 [upstream limit], ST46213032 [downstream limit]) on 5 days (11th, 14th, 20th, 24th, 27th April 2021) using landing nets (51 cm, 2–5 mm mesh, Drennan). Water depth was ~50–100 cm, although was 5–10 cm in two capture locations, and the river width was ~7.2 m. Three turbidity measurements from three separate river water samples were taken in the morning of each day using a Thermo Scientific Orion AQ310 turbidity meter measuring Nephelometric Turbidity Units (NTU) and calibrated with known turbidity standards (0, 20, 100 NTU) (Table A1). While lentic ecosystems such as ponds, lakes and marshes trap excess nutrients that can lead to algal‐based turbidity (Schindler, 2006), turbidity due to inorganic sediments is typical of lotic river systems like our study site. 12 or 16 fish with no visible signs of being in breeding condition were captured on each day. Following capture, the fish were transferred to a black bucket (14.5 L) filled with river water and aerated with an airstone (POPETPOP Aquarium Air Pump USB Powered). The bucket was covered with netting to protect the fish from aerial predators and kept in the shade prior to the start of the trials.

### 2.3 Experimental protocol

**FIGURE 1:**
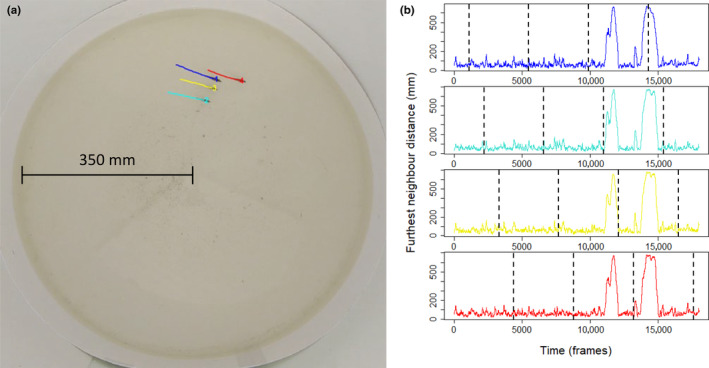
Image of the experimental arena and a group of four fish during a turbid trial. Coloured trajectories are generated by the movement tracking software idTracker (a). Right: Time series of furthest neighbour distance from the trial pictured in the left image where each colour corresponds to the coloured trajectories of the fish in the left image (b). Dashed black lines show where the time series were sampled using the staggered downsampling of the data to maximise independence of the data.

Trials took place in a white circular acrylic glass arena (70 cm ⌀, Figure 1a) surrounded by a 40 cm high wall of white matt foamed PVC sheet (1 mm thickness) to minimise visual disturbance. The arena was filled with river water to 5 cm depth, consistent with the vertical plane depth of stickleback shoals observed in the wild near the water surface (Ward et al., 2017). During trials, fish were filmed from above with a GoPro Hero 6 waterproof action camera in linear view with 2.7 K resolution (2704 × 1520 pixels) at 30 frames per second. The camera was mounted downward facing and perpendicular to the water surface on a tripod, 75 cm above the base of the arena. The setup was surrounded by a white shower curtain to diffuse light and minimise reflections on the water surface.

**FIGURE A1:**
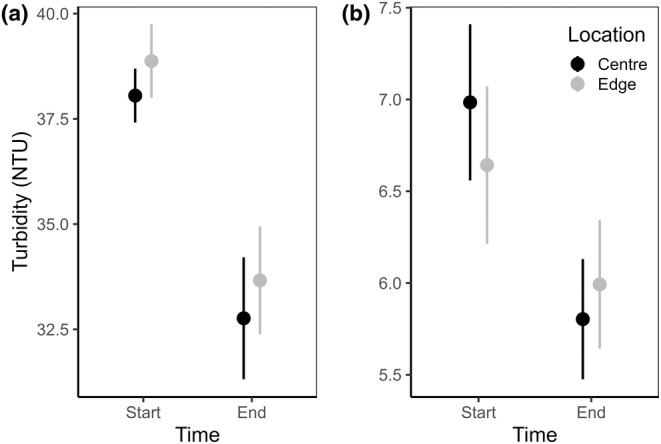
Change in turbidity between the start and end of trials for (a) the turbid and (b) the clear treatments. Colours represent whether the samples came from the centre (black) or edge (grey) of the arena and points depict the mean turbidity levels and lines show the standard error of the mean in each case.

Fifteen minutes before the start of the first trial for a group, four individuals were haphazardly selected from the black bucket with a hand net and transferred to an identical holding bucket containing fresh river water. Two trials were conducted per group, one clear water treatment and one turbid water treatment. The treatment order (1st or 2nd) was alternated between consecutive groups, and the first treatment for the first group tested in a day was alternated between days. For the turbid treatment (35.8 ± 5.3 NTU, mean ± SD), turbidity was generated by adding fine kaolin clay powder (Mystic Moments, Superfine British Clay) to the river water and mixing with a cane. For the clear treatment (6.4 ± 1.7 NTU, mean ± SD), no clay was added but the water was mixed as above to disperse any naturally occurring sediment. Turbidity below 10 NTU is typical of levels recorded for the River Cary, however 35 NTU is within the range of recorded turbidity levels (https://environment.data.gov.uk/water‐quality/) and is below the levels that are known to cause physiological stress in fish (e.g. Hasenbein et al., 2016; Sutherland & Meyer, 2007). Directly before and after each trial, turbidity measurements were recorded from the centre and at the edge of the arena (Figure A1). Although there was a significant effect of time (start or end of trial) on turbidity in both the clear (linear model (LM): *F*_1,71_ = 6.28, *p* = .015) and turbid (LM: *F*_1,71_ = 24.46, *p* < .001) treatment, which was expected due to suspended solids settling during the trials, there was no significant effect of test order within day (clear: LM: *F*_1,71_ = 3.76, *p* = .057, turbid: LM: *F*_1,71_ = 3.33, *p* = .072) or day (clear: LM: *F*_1,71_ = 1.75, *p* = .19, turbid: LM: *F*_1,71_ = 0.040, *p* = .84). We therefore treated treatment as a categorical variable (clear or turbid) in our analyses.

**FIGURE A2:**
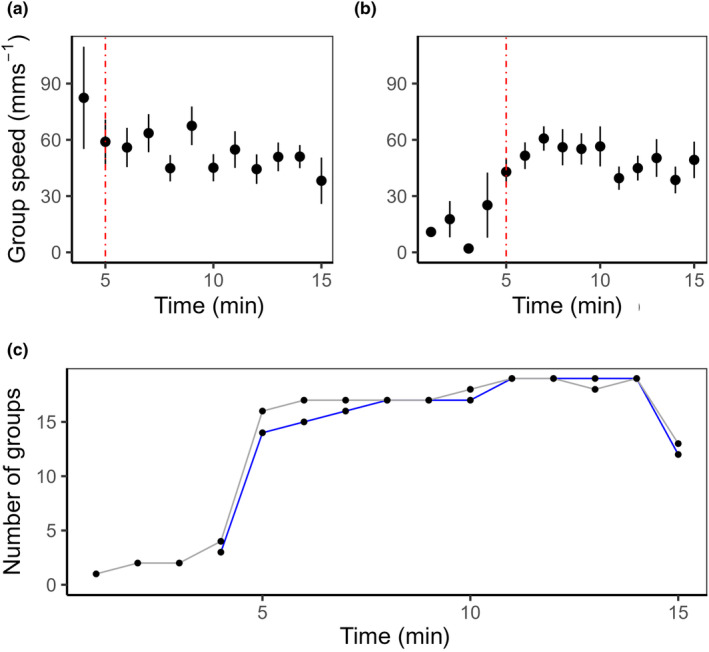
Mean and standard error of group centroid speed (mms^−1^) at 1 minute time intervals for the clear (a) and turbid (b) treatment. The red dashed line marks the end of the 5‐minute acclimatisation period and the start of the trial. The number of groups with complete tracking (i.e. all individuals in the group are identified by the tracking software in this frame and the two previous frames) at each time interval are displayed in the figure below with the clear treatment shown in blue and turbid treatment in grey (c). A step change in number of groups occurs at 5 min, indicative of individuals habituating to the arena and becoming active.

At the start of a trial, the group of four were netted into the centre of the arena. The curtain surrounding the setup was then closed and the video recording was started remotely. Each trial lasted for 15 min including a 5‐min acclimatisation period that was not included in data analyses. Our decision to limit the length of acclimatisation to 5 min and trials to 10 min was, in part, because our study was aimed at understanding the effects of short‐term rather than chronic exposure to turbidity and also because the kaolin clay powder settled on the base of the arena, reducing turbidity over time (Figure A1). In a previous study, groups of sticklebacks took a median of between 20 and 35 s for the first individual to leave a refuge in turbid (~31NTU) and clear water (Chamberlain & Ioannou, 2019), and although in our experiment there was no refuge, this is well within our acclimatisation period. Furthermore, our tracking data indicate that individuals were typically active by 5 min and the mean group speed was stable during the trial, suggesting that making the acclimatisation period longer would not have an effect on the results (Figure A2). At the end of the first trial for each group, the group was transferred back to their holding bucket for ~35 min and the arena water was changed (and the turbidity treatment generated for turbid treatments). At the end of the second trial, fish were returned to the holding bucket and immediately released. Trials were conducted between 13:00 and 18:40 in ambient light conditions.

### 2.4 Data processing

Fish in the videos were tracked using idTracker and tracking quality was manually checked by visual inspection using idPlayer (Perez‐Escudero et al., 2014). We applied a maximum speed filter for the fish (60 pixels per frame, approximating the maximum burst swimming speed reported for freshwater sticklebacks [Taylor & McPhail, 1986]) and whenever at least one fish exceeded the threshold in a frame, all data were removed from that frame to exclude tracking errors. The cartesian coordinates of each fish measured in pixels were then smoothed over the remaining consecutive frames using a Savitzky–Golay filter (span 15, degree 3) to further reduce noise from inaccurate tracking. Where there were missing coordinates for any fish in the group for a frame or the two frames preceding it, all data were removed from that frame because we could not reliably calculate the response measures.

### 2.5 Response measures

We calculated two measures of social cohesiveness for each individual in each frame: the distance between the centroid of a focal individual and that of their closest neighbour (the nearest neighbour distance) and the distance between the centroid of a focal individual and that of their furthest neighbour (the furthest neighbour distance). We calculated the heading difference between individuals and their nearest neighbour as a measure of coordination in directional movement, and the bearing angle of the nearest neighbour to the individuals to quantify the relative position of fish. A bearing angle of 0 degrees represents a neighbour directly in front of the focal fish, and 90 degrees and 180 degrees are directly to the side and behind, respectively. The bearing angles were rescaled to range between 0° (the neighbour directly ahead or behind) and 90° (the neighbour directly alongside the focal fish) so individuals ahead and behind the focal individuals were treated similarly in analyses (Ginnaw et al., 2020). For analyses, distances were converted to units of mm, and speeds to units of mm per second, using the known internal diameter of the arena and its measurement in pixels from still images from the videos. This showed some small variation between days and trials due to movements in the camera position. The fish's body length measured from video stills did not differ significantly between days of the experiment (LM: *F*_1,74_ = 0.36, *p* = .55). Note that each fish had two body length estimates, one from the clear treatment and one from the turbid treatment, but there was not sufficient variation between individuals within groups to use this as a method to identify individuals between the two trials per group.

### 2.6 Statistical analyses

To analyse the effects of turbidity on nearest neighbour distance, furthest neighbour distance, heading difference with nearest neighbour, bearing of nearest neighbour and speed while accounting for temporal autocorrelation in each variable (measured for each individual in each frame of the videos), we downsampled the trajectory data of each individual by the maximum time lag to achieve zero autocorrelation for any individual fish for any of the five variables. This was 4347 frames for nearest neighbour distance, equivalent to 145 s, which we rounded up to 4400 frames. Rather than sample the same frames for all fish in a trial, we then staggered the downsampling of frames between fish to minimise the correlations between individuals and maximise independence of the data. We therefore sampled the following frames: fish 1 was sampled at 1100, 5500, 9900, 14,300; fish 2 at 2200, 6600, 11,000, 15,400; fish 3 at 3300, 7700, 12,100, 16,500; fish 4 at 4400, 8800, 13,200, 17,600 (Figure 1b), where the identities of the fish were randomly assigned by idtracker for each trial. In the 37 of 608 cases where no data were available at the selected time point, we found the nearest time point in a 450 frame (15 s) window either side of the sample frame as a replacement. This resulted in one replacement. In the other 36 cases, no data were available in the time window.

Because knowledge of the identities of the fish could not be maintained between the two trials for each group, we were unable to account for repeated measures of individuals between treatments. For analyses, we created a variable of fish identity to give each fish within a group within a treatment a unique identity. Each model then included this fish identity variable nested within group identity as a random effect. Using the downsampled data, we first tested whether individuals' swimming speeds differed between the treatments using a linear mixed model (LMM) with treatment, treatment order (1st or 2nd) and body length as main effects. For tests of the effects of treatment on nearest neighbour distance, furthest neighbour distance, heading difference with nearest neighbour and bearing angle to nearest neighbour, we used LMMs with treatment, treatment order and body length as main effects. We initially included a treatment × treatment order interaction term in the models to test whether the effects of turbidity were stronger when fish where newly introduced to the experiment. This interaction term was not significant (*p* > .05) in all cases and subsequently excluded from the final analyses. We ran the models for nearest neighbour distance, furthest neighbour distance, heading difference and bearing angle with and without swimming speed as a main effect to establish whether speed had a mediating effect on the behavioural responses to treatment.

**FIGURE 2:**
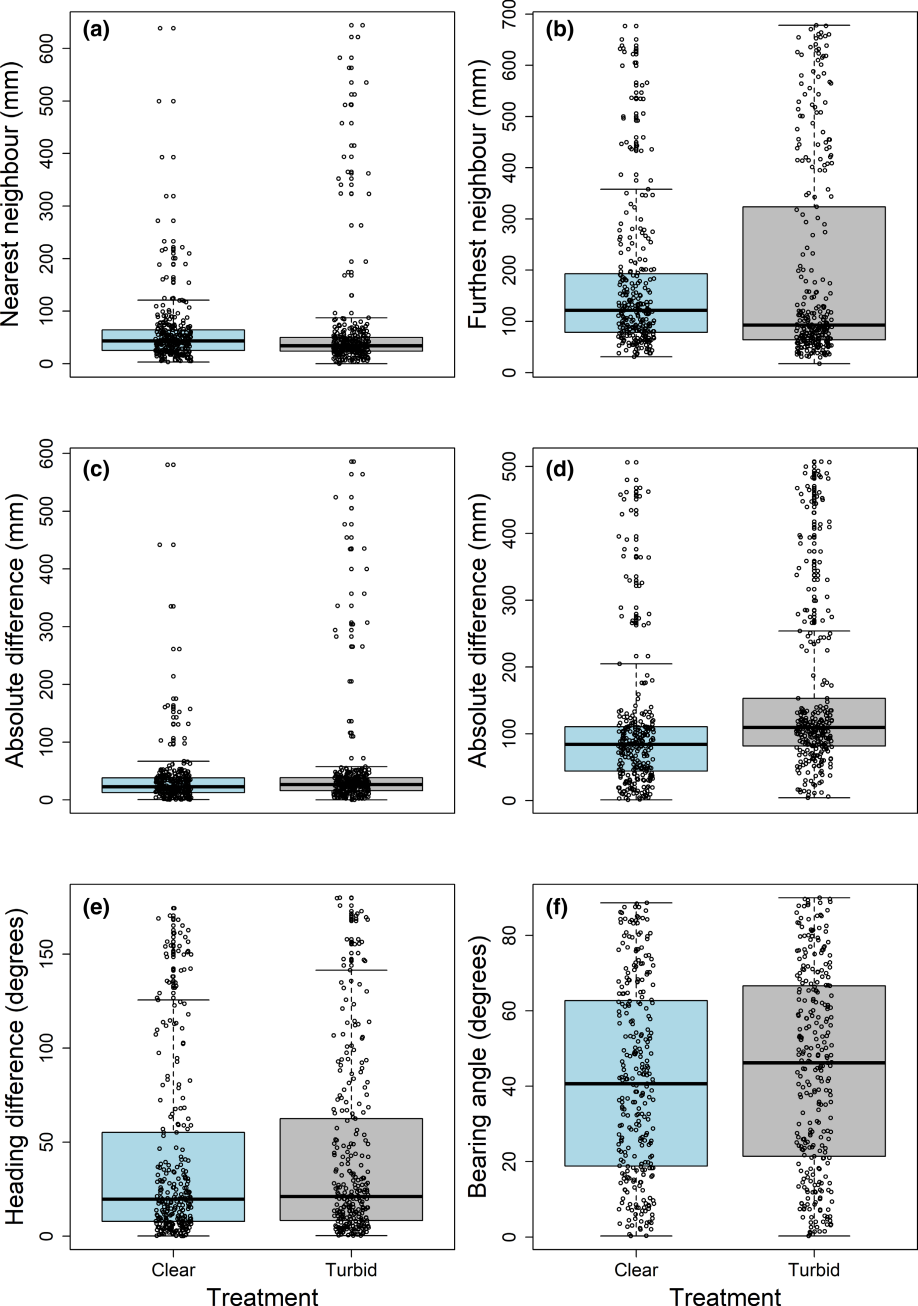
Effect of turbidity on the nearest neighbour distance (a), furthest neighbour distance (b), absolute difference between nearest neighbour distance and mean nearest neighbour distance for that treatment (c), absolute difference between furthest neighbour distance and mean furthest neighbour distance for that treatment (d), heading difference with the nearest neighbour (e) and bearing angle to nearest neighbour (rescaled 0–90 degrees) (f). Boxplots in blue display data for the clear treatment and boxplots in grey display data for the turbid treatment. The median across individual fish is shown by the solid lines, the interquartile range is enclosed within the box, and the whiskers extend to the most extreme data point within 1.5× the interquartile range. Circles display the raw data from the downsampled data set of four data points per individual, which achieved temporal independence.

Visual inspection of the distributions of the data for nearest neighbour and furthest neighbour distance showed some evidence of differences in the variability of the data (Figure 2a,b). To test whether the variability in the nearest and furthest neighbour distances differed between the two treatments, we subtracted the treatment mean from each value. Absolute differences from the treatment mean for nearest neighbour and furthest neighbour distances were then analysed as response variables in LMMs including treatment, treatment order and body length as main effects and with and without speed as a main effect. Group identity was excluded as a random effect from models of variability in nearest neighbour distance due to the variance between groups being estimated as zero.

Statistical analyses were carried out in R (version 4.3.0, https://www.R‐project.org/). All LMMs were performed with restricted maximum likelihood in R package lme4 (version 1.1–33, Bates et al., 2015). The significance of effects was tested with Kenward‐Roger *F*‐tests in package lmerTest (version 3.1‐3, Kuznetsova et al., 2017). Model residual diagnostics were checked using R package DHARMa (version 0.4.6, Hartig, 2022) by graphically plotting simulated model residuals (*n* = 1000 simulations). We adopted a discretionary approach to identify model misspecification (Hartig, 2022). All response variables except for speed and the bearing to nearest neighbour were log_10_ transformed to improve the fit of the residuals to assumptions of normality of residuals and homogeneity of variances.

## 3 RESULTS

**FIGURE 3:**
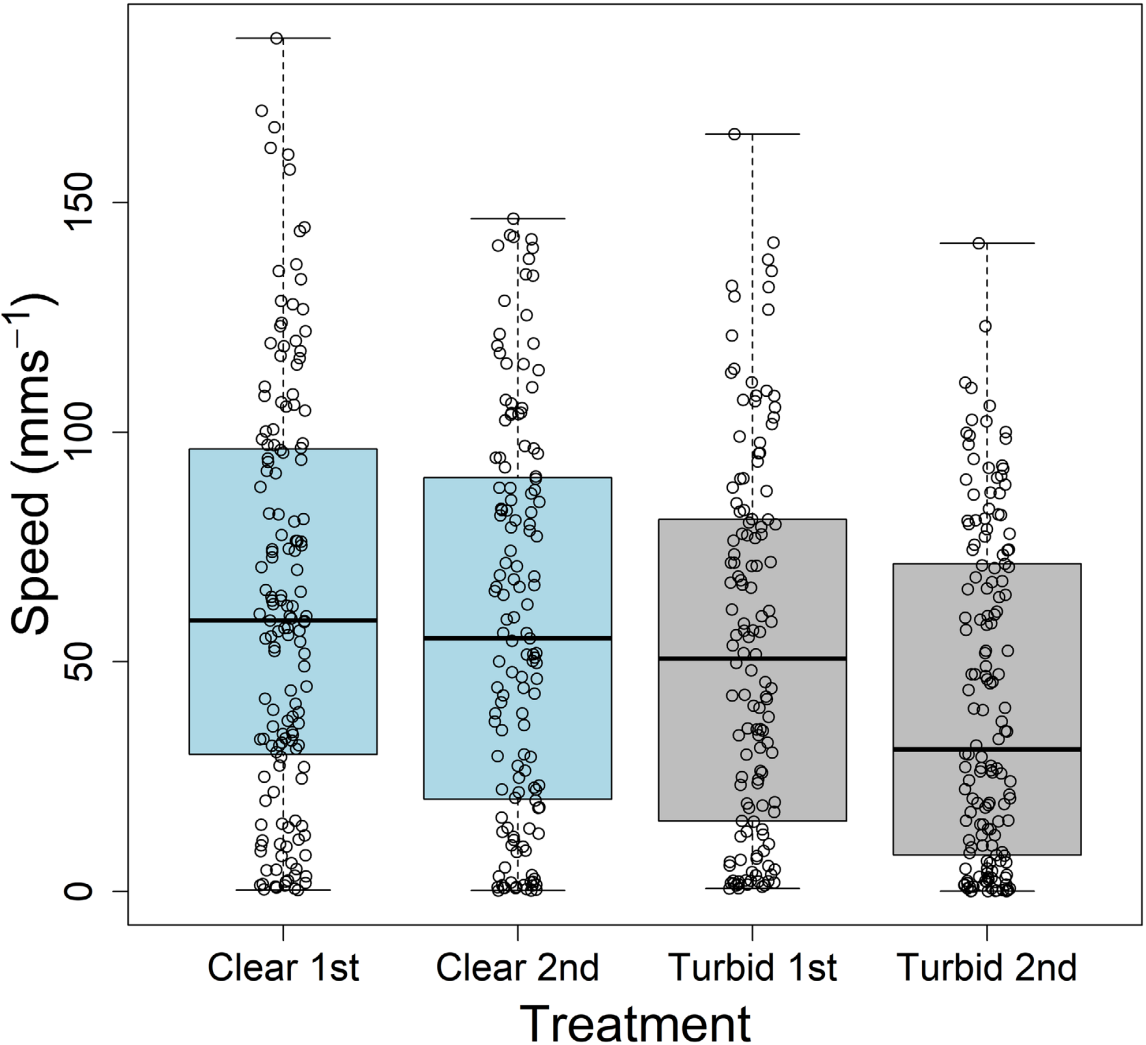
Effect of turbidity and treatment order (1st or 2nd) on the swimming speed of individual fish. Boxplots in blue display data for the clear treatment (‘C’) and boxplots in grey display data for the turbid treatment (‘T’). The median across individual fish is shown by the solid lines, the interquartile range is enclosed within the box, and the whiskers extend to the most extreme data point within 1.5 × the interquartile range. Circles display the raw data from the downsampled data set of four data points per individual, which achieved temporal independence.

The fish swam significantly slower in the turbid compared to the clear treatment (Table 1, Figure 3). Fish were significantly closer to their nearest neighbour in the turbid compared to the clear treatment (Figure 2a), but speed had a modulating effect where the effect of treatment became marginally insignificant when speed was included as a main effect in the model (Table 1). There was also evidence of fish acclimatising to the experiment with slower swimming speed and increased nearest neighbour distance for individuals in the second treatment compared to the first treatment, irrespective of which treatment was first (Table 1, Figure 3). Furthest neighbour distances did not differ significantly between the treatments (Table 1, Figure 2b). However, both nearest and furthest neighbour distance were more variable in the turbid treatment, as the absolute differences from the treatment means were significantly different between treatments (Table 2, Figure 2c,d). The effect of treatment on the variability in the nearest neighbour distance became non‐significant when speed was included as a main effect, and speed was significantly negatively associated with the variability in the nearest neighbour distance (Table 2). Speed was not associated with the variability in the furthest neighbour distance, however, which remained more variable in the turbid treatment even when speed was included as a main effect (Table 2). The heading difference to the nearest neighbour was smaller at faster swimming speeds, that is fish were more aligned when swimming faster, however there was no evidence that turbidity altered the alignment with, or relative bearing to, the nearest neighbour (Table 1, Figure 2e,f).

**TABLE 1:** Results of linear mixed models testing for the effects of treatment on the behaviours of individuals in groups using Kenward‐Roger *F*‐tests.

Response variable	Explanatory variable	Estimate	Standard error	Degrees of freedom	*F*‐value	*p‐*value
Speed (mms^−1^)	**Treatment**	−8.89	4.08	130.67	4.75	.031
	**Treatment order**	−13.88	4.08	130.82	11.57	.001
	Body length (mm)	0.34	0.67	137.45	0.24	.63
Nearest neighbour distance (mm)	**Treatment**	−0.077	0.036	131.15	4.64	.033
	**Treatment order**	0.089	0.036	130.98	6.26	.014
	Body length (mm)	0.003	0.006	143.01	0.28	.60
Nearest neighbour distance (mm)	Treatment	−0.069	0.035	131.95	3.81	.053
	**Treatment order**	0.10	0.035	135.62	8.30	.005
	Body length (mm)	0.002	0.006	142.86	0.21	.64
	**Speed (mms** ^**−1**^ **)**	0.001	0.000	562.21	5.62	.017
Furthest neighbour distance) (mm)	Treatment	−0.013	0.029	131.67	0.20	.66
	Treatment order	−0.029	0.029	131.51	1.02	.31
	Body length (mm)	0.002	0.005	135.70	0.11	.75
Furthest neighbour distance) (mm)	Treatment	−0.013	0.029	132.31	0.19	.67
	Treatment order	−0.029	0.030	136.47	1.00	.33
	Body length (mm)	0.002	0.005	135.31	0.10	.75
	Speed (mms^−1^)	0.000	0.000	548.28	0.012	.91
Heading difference with nearest neighbour (degrees)	Treatment	0.031	0.054	131.15	0.34	.56
	Treatment order	0.071	0.054	130.96	1.73	.19
	Body length (mm)	−0.005	0.009	138.40	0.27	.61
Heading difference with nearest neighbour (degrees)	Treatment	−0.019	0.049	132.02	0.15	.70
	Treatment order	−0.015	0.050	136.31	0.091	.76
	Body length (mm)	−0.004	0.008	142.12	0.18	.67
	**Speed (mms** ^**−1**^ **)**	−0.006	0.001	550.55	102.70	<.001
Bearing angle to nearest neighbour (rescaled 0–90 degrees)	Treatment	2.57	2.19	130.73	1.38	.24
	Treatment order	3.32	2.19	130.52	2.30	.13
	Body length (mm)	−0.094	0.34	121.06	0.071	.79
Bearing angle to nearest neighbour (rescaled 0–90 degrees)	Treatment	2.40	2.21	131.32	1.18	.28
	Treatment order	3.03	2.24	135.49	1.85	.18
	Body length (mm)	−0.086	0.35	119.59	0.059	.81
	Speed (mms^−1^)	−0.021	0.027	522.73	0.62	.43

*Note*: Each row gives the *p‐*value for a different explanatory variable in the model. Statistically significant variables are highlighted in bold. The clear treatment is the reference level for the variable treatment.

**TABLE 2:** Results of linear mixed models testing for the effects of treatment on the variability in nearest neighbour and furthest neighbour distance using Kenward‐Roger *F*‐tests.

Response variable	Explanatory variable	Estimate	Standard error	Degrees of freedom	*F*‐value	*p*‐value
Absolute difference between nearest neighbour distance and mean nearest neighbour distance for that treatment (mm)	**Treatment**	0.010	0.28	145.43	4.55	.035
	Treatment order	0.028	0.047	145.59	0.36	.55
	Body length (mm)	−0.076	0.047	143.68	0.011	.92
Absolute difference between nearest neighbour distance and mean nearest neighbour distance for that treatment (mm)	Treatment	0.089	0.047	146.09	3.56	.061
	Treatment order	0.010	0.047	149.39	0.041	.84
	Body length (mm)	0.000	0.007	143.26	0.004	.95
	**Speed (mms** ^**−1**^ **)**	−0.001	0.000	562.51	7.07	.008
Absolute difference between furthest neighbour distance and mean furthest neighbour distance for that treatment (mm)	**Treatment**	0.23	0.037	145.07	37.46	<.001
	Treatment order	−0.036	0.037	145.13	0.97	.33
	**Body length (mm)**	0.014	0.006	143.12	5.97	.016
Absolute difference between furthest neighbour distance and mean furthest neighbour distance for that treatment (mm)	**Treatment**	0.23	0.037	145.51	36.72	<.001
	Treatment order	−0.038	0.037	149.09	1.03	.31
	**Body length (mm)**	0.014	0.006	142.60	5.98	.016
	Speed (mms^−1^)	0.000	0.000	551.99	0.082	.77

*Note*: Variability is quantified by the absolute difference between nearest/furthest neighbour distance and mean nearest/furthest neighbour distance for that treatment. Each row gives the *p‐*value for a different explanatory variable in each model. Statistically significant variables are highlighted in bold. The clear treatment is the reference level for the variable treatment.

## 4 DISCUSSION

Previous experimental studies on freshwater fish have demonstrated reduced cohesion and coordination in fish shoals under poor visual conditions, including in three‐spined sticklebacks (Chamberlain & Ioannou, 2019; Ginnaw et al., 2020). In contrast, in our experiment we found that sticklebacks showed enhanced cohesion as measured by closer nearest neighbour distance under turbid conditions (~35NTU), consistent with studies in marine species (Ohata et al., 2014) and sticklebacks in dynamic visually noisy environments (Matchette & Herbert‐Read, 2021). However, our statistical analysis revealed that this effect could be accounted for by a reduction in swimming speed in turbid water, which then had the effect of reducing nearest neighbour distance. Empirical and theoretical studies of collective behaviour have established that changes in individuals' speeds are a key mechanism for changes to the collective behaviour of fish shoals (Herbert‐Read et al., 2011; Jolles et al., 2020; Katz et al., 2011; Schaerf et al., 2017). It is unclear from our experiment whether fish reduce their swimming speed in turbid water as an adaptive response, for example to maintain nearest neighbour distance (and associated benefits for social information detection) in low visibility, or whether this change occurs as a by‐product of other effects of the turbid environment, such as increases in the perception of predation risk or as a stress response (Ajemian et al., 2015; Chamberlain & Ioannou, 2019). While it is possible that physiological effects such as changes in respiration could play a role in reducing swimming speeds (e.g. Berli et al., 2014), our treatment exposure was short and our treatment level of turbidity was below that showing strong physical or physiological effects in fish (e.g. Hasenbein et al., 2016; Sutherland & Meyer, 2007). For example, Spotfin chub reared in different levels of sedimentary turbidity did not show an effect of turbidity on gill condition until 100 mg L^−1^ suspended sediment concentration. While the effect of turbidity on the nearest neighbour distance was indirect, and there was no evidence that the furthest neighbour distance increased or decreased on average in the turbidity treatment, neighbour distances were more variable in turbid water. The nearest neighbour distance was less variable as swimming speeds increased, and the effect of turbidity on the variability in nearest neighbour distance became non‐significant when swimming speed was factored into the model. However, there was no association of furthest neighbour distance with swimming speed, and the furthest neighbour distance was more variable in turbid water even when swimming speed was included in the model. Together, these results are consistent with cohesion of the group as a whole being less stable in turbid water, where groups are usually compact but occasionally spread out over a wide area. In contrast, the fish were better able to maintain the distance to their nearest neighbour. This supports experimental work on guppies showing that shoals become more fragmented in turbid water (Kimbell & Morrell, 2015a). The functional significance of group fragmentation in the wild may include increased exposure to predation risk (Ioannou et al., 2019) and negative effects associated with a reduction in the availability of social information (e.g. a reduction in the speed in locating food resources, Pitcher et al., 1982). That fragmentation appeared to be short‐lived in our experiment may be due to the small size of our arena. Under natural conditions, fish separated from the main group may not be able to regain contact so easily and hence will lose the benefits of group living entirely. This could be particularly detrimental if fish rely even more on social information in turbid environments because access to private visual information is compromised.

We did not find evidence that the fish modified their alignment to their nearest neighbour or their relative spatial positioning in turbid water as measured by the bearing of their nearest neighbour. An experiment on groups of 4 three‐spined sticklebacks in darkness found that the fish increased their heading differences with nearest neighbours compared to control conditions (Ginnaw et al., 2020). An absence of this effect in our experiment may be because the reduction in visibility due to turbidity was not as extreme, so the fish were able to maintain contact with their nearest neighbour under turbidity and hence resist changes to interactions with this neighbour. That the individuals did not significantly modify their bearing angle to their nearest neighbour suggests that turbidity did not strongly disrupt leadership behaviour and decision‐making, which is weighted towards individuals positioned at the front of moving groups in this species (Bevan et al., 2018; Harcourt et al., 2009; Nakayama et al., 2012).

Changes over time in collective behaviour have been shown repeatedly in fish shoals (Ioannou & Laskowski, 2023; MacGregor & Ioannou, 2021; Miller & Gerlai, 2012). In our study, we observed that fish became further from their nearest neighbour in the second trial compared to the first, and also swam less rapidly, but there was no effect of trial order on the other response variables. The lack of statistically significant interactions between the treatment and testing order in our study suggests that over this time scale, processes such as acclimatisation to the testing setup were not affected by whether the water was clear or turbid. Over longer time scales, it is unclear whether the treatment differences would have persisted, for example, if fish in the turbid treatment increased their swimming speed after overcoming an initial period of neophobia, this could result in convergence in behaviour between the treatments. How organisms change their behaviour in turbid water over variable time scales is not well understood but may have key applied importance in whether and how they can adapt to changing environmental conditions.

Our study demonstrates that key aspects of collective behaviour can be quantified under controlled conditions in a field setting. That our experimental setup allows for the study of species that are not amenable to laboratory conditions opens up the possibility of future work comparing shoaling responses to turbidity across a wide range of species that may differ in their sensory morphology and, hence, sensitivity to the effects of turbidity. Nevertheless, there are some limitations to our experimental approach, for example, we could not control environmental noise such as fluctuations in lux levels with cloud cover within the arena. There were also constraints on the size of our arena to make it portable, which would limit future studies using a similar setup to small species and juveniles of larger species (although shoaling tendency tends to be stronger in juveniles than in adults [Shaw, 1978]). A next step will be to test whether shoaling responses to turbidity change between populations depending on their natural turbidity regimes. This will reveal the generality of our findings and yield further insights into the capacity of shoaling fish to respond adaptively to turbidity.

## AUTHOR CONTRIBUTIONS

**Hannah E. A. MacGregor:** Conceptualization (equal); data curation (lead); formal analysis (equal); funding acquisition (lead); project administration (lead); writing – original draft (lead); writing – review and editing (equal). **Christos C. Ioannou:** Conceptualization (equal); formal analysis (equal); writing – original draft (supporting); writing – review and editing (equal).

## FUNDING INFORMATION

This work was funded by an Association for the Study of Animal Behaviour Research Grant awarded to HEAM.

## CONFLICT OF INTEREST STATEMENT

The authors declare no conflict of interest.

## Data Availability

The data set and code accompanying this paper can be downloaded from Dyrad Digital Repository: https://doi.org/10.5061/dryad.bk3j9kdjp. Reviewer sharing link before acceptance: https://datadryad.org/stash/share/d_‐BKg09wPT‐3_u_xEyvQWWJAcCrkFNgT7n8cbLCf80.

## APPENDIX 1

**TABLE A1:** Turbidity readings for the River Cary taken during the experiment. River water was clear (<10 NTU) on all occasions.

Date	Turbidity (mean ± SD)	Temperature (°C)	Study day	Number of groups tested
28/03/2021	8.2 ± 0.53	–	Pilot	–
03/04/2021	5.7 ± 0.44	–	Pilot	–
11/04/2021	5.5 ± 0.17	9.14	Day 1	4
14/04/2021	3.8 ± 0.35	10.46	Day 2	4
20/04/2021	4.4 ± 0.69	11.24	Day 3	4
24/04/2021	3.6 ± 0.31	15.96	Day 4	4
27/04/2021	5.9 ± 0.83	13.68	Day 5	3
